# Gait Analyses in Mice: Effects of Age and Glutathione Deficiency

**DOI:** 10.14336/AD.2017.0925

**Published:** 2018-08-01

**Authors:** J. Thomas Mock, Sherilynn G Knight, Philip H Vann, Jessica M Wong, Delaney L Davis, Michael J Forster, Nathalie Sumien

**Affiliations:** Department of Pharmacology & Neuroscience, Center for Neuroscience Discovery, Institute for Healthy Aging, University of North Texas Health Science Center at Fort Worth, Fort Worth, TX, 76107 USA.; Department of Pharmacology & Neuroscience, Center for Neuroscience Discovery, Institute for Healthy Aging, University of North Texas Health Science Center at Fort Worth, Fort Worth, TX, 76107 USA.; Department of Pharmacology & Neuroscience, Center for Neuroscience Discovery, Institute for Healthy Aging, University of North Texas Health Science Center at Fort Worth, Fort Worth, TX, 76107 USA.; Department of Pharmacology & Neuroscience, Center for Neuroscience Discovery, Institute for Healthy Aging, University of North Texas Health Science Center at Fort Worth, Fort Worth, TX, 76107 USA.; Department of Pharmacology & Neuroscience, Center for Neuroscience Discovery, Institute for Healthy Aging, University of North Texas Health Science Center at Fort Worth, Fort Worth, TX, 76107 USA.; Department of Pharmacology & Neuroscience, Center for Neuroscience Discovery, Institute for Healthy Aging, University of North Texas Health Science Center at Fort Worth, Fort Worth, TX, 76107 USA.; Department of Pharmacology & Neuroscience, Center for Neuroscience Discovery, Institute for Healthy Aging, University of North Texas Health Science Center at Fort Worth, Fort Worth, TX, 76107 USA.

**Keywords:** Aging, glutathione deficiency, gait, speed, catwalk

## Abstract

Minor changes (~0.1 m/s) in human gait speed are predictive of various measures of decline and can be used to identify at-risk individuals prior to further decline. These associations are possible due to an abundance of human clinical research. However, age-related gait changes are not well defined in rodents, even though rodents are used as the primary pre-clinical model for many disease states as well as aging research. Our study investigated the usefulness of a novel automated system, the CatWalk™ XT, to measure age-related differences in gait. Furthermore, age-related functional declines have been associated with decreases in the reduced to oxidized glutathione ratio leading to a pro-oxidizing cellular shift. Therefore the secondary aim of this study was to determine whether chronic glutathione deficiency led to exacerbated age-associated impairments. Groups of male and female wild-type (gclm^+/+^) and knock-out (gclm^-/-^) mice aged 4, 10 and 17 months were tested on the CatWalk and gait measurements recorded. Similar age-related declines in all measures of gait were observed in both males and females, and chronic glutathione depletion was associated with some delays in age-related declines, which were further exacerbated. In conclusion, the CatWalk is a useful tool to assess gait changes with age, and further studies will be required to identify the potential compensating mechanisms underlying the effects observed with the chronic glutathione depletion.

Aging is associated with a decline in overall motor function across various species, including rodents and humans [1-4]. Interestingly, human gait speed can be utilized to stratify risk of decline in cognitive function, mobility, loss of independence, increased fall risk, institutionalization, as well as overall mortality risk [5]. This association of gait speed with many dimensions of aging has been found consistently in humans, where minor changes (~0.1 m/s) in gait speed were predictive of various measures of decline and can be used to identify at-risk individuals prior to further decline [6]. While human gait research is abundant, age-related gait changes in rodents are not well defined even though they are used as the primary pre-clinical model for many disease states and aging research [7].

Although rodent gait changes as a result of aging are not as well characterized as changes in human gait, research on other aspects of motor function in rodents has shown age-related decreases in spontaneous movement speed [2, 3, 8], physical activity [9, 10], as well as balance and motor coordination [2]. Importantly, both human and mouse functional motor decline follow similar patterns, where motor function decline occurs earlier in life than other more debilitating outcomes, highlighting the importance of motor function as both a clinical and preclinical measure [9, 11]. Indirect measures of gait speed through open field [12] or balance beam testing [13] hinted at age-related slowing of gait speed, but with methodological limitations as gait analysis was not the primary measure and no other gait variables were recorded. Direct studies of rodent gait via treadmill analysis at a set speed have shown age-related changes, however studies in mice and humans have demonstrated that treadmill and free gait parameters are not equivalent [14, 15]. Furthermore, most gait analyses in rodents have focused on changes across the first 12 months of life, which equates to adult growth along with aging. This presents an opportunity to determine quantitative relationships between gait changes and age across the lifespan of free-running rodents. A relatively novel way to measure gait is to use the CatWalk™ XT, an automated system using a sophisticated software allowing rodents to walk in a low-stress environment allowing gait analyses in a low-stress environment. Thus far, the CatWalk has primarily been validated in modeled disease states including traumatic brain injury, stroke, spinal cord injury, Parkinson’s disease, osteoarthritis, and ataxia [16-20]. However, to date there have been no studies on the effect of age in mice on the qualitative and quantitative gait outcomes using this apparatus.

Additionally, the redox stress theory of aging postulates that while a moderate level of reactive oxygen species (ROS) is biologically useful for cell signaling, an overabundance of ROS can lead to accumulation of oxidative damage and a pro-oxidizing shift in reduction-oxidation (redox) state preceding cellular dysfunction [21, 22]. This pro-oxidant shift in aging is primarily measured via changes in the ratio of reduced to oxidized glutathione (GSH:GSSG), where aging leads to a decline in the GSH:GSSG ratio indicating less redox potential [23]. Although several redox couples interact with GSH to maintain overall cellular redox state, the glutathione redox couple (GSH:GSSG) is the most abundant, and as such modulation of redox status can be achieved by altering levels of GSH [24]. Synthesis of GSH occurs in two subsequent enzymatic reactions, formation of y-glutamylcysteine (y-GC) from glutamate and cysteine via glutamate cysteine ligase followed by conversion of y-GC to GSH via GSH synthetase [25]. The synthesis of GSH is rate-limited by the glutamate cysteine ligase (gcl) enzyme, a heterodimer consisting of a catalytic (gclc) and modifier (gclm) subunit [25]. While gclc contains all catalytic capacity, gclm increases the V_max_ and the affinity for glutamate, and decreases feedback inhibition from GSH [26]. While homozygous knockout of gclc is embryonic lethal, global knockout of the gcl modifier subunit (gclm^-/-^) in mice leads to a 70-90% decrease in GSH levels across various tissues, including liver, brain, kidney, and lung [27]. Adult gclm^-/-^ mice present with chronically decreased levels of GSH, are more susceptible to oxidative insults [28], and have a more pro-oxidative redox cellular environment [27]. These changes in gclm^-/-^ mice could lead to an early aging phenotype if a more oxidative redox state is in fact a determinant factor in aging.

Accordingly, the objectives of this study were to validate the Catwalk™ XT in assessment of age-related gait changes and determine if chronic glutathione depletion exacerbated these gait variations. To achieve the goals, male and female gclm^-/-^ and gclm^+/+^ mice were tested on the Catwalk at 3 different target ages (4, 10 and 17 months). The operational hypothesis was that gait measures would decline with advanced age and that impairments would occur earlier in glutathione depleted mice.

## MATERIALS AND METHODS

### Animals

Procedures were approved by the Institutional Animal Care and Use Committee at the University of North Texas Health Science Center at Fort Worth, and adhered to NIH guidelines. The mice heterozygous for gclm were generated on a C57BL/6J (B6.129) background, acquired from Dr. Terence Kavanaugh, rederived and backcrossed at least 7 generations into C57BL/6 mice by Jackson Laboratories. Once in the UNT Health Science Center vivarium, triads of gclm^+/-^ were mated to obtain wild-type (gclm^+/+^) and knock-out (gclm^-/-^) littermates. From the in-house breeding colony, male and female mice were housed and aged in groups of 2-4, separated according to sex and genotype, in standard polycarbonate cages (28 x 17 x 12.5 cm) with corncob bedding and *ad libitum* access to water and standard rodent chow (LabDiet® R&M 5LG6 5S84; catalog number: 1813505 from TestDiet, Richmond, IN), and were maintained at ambient temperature (23 ± 1° C), under a 12-h light/dark cycle starting at 0600. At the target ages of 4, 10, and 17 months for this study, squads of mice from the aging colony were used for gait analyses (Table 1) in a cross-sectional design.

### Quantitative gait analyses

Gait measures were determined using the CatWalk™ XT system (Noldus Information Technology, The Netherlands). The CatWalk is an apparatus used for semi-automated objective rodent gait analysis via video recording. Importantly, this system allows for the animals to voluntarily move at preferred speeds in a similar fashion to clinical gait testing in humans. The device consists of (i) a 130 cm long hardened glass platform with an adjustable alleyway to limit movements to straight lines, (ii) a red overhead light, (iii) a green LED light attached to the glass platform, and (iv) a high speed color camera mounted below the platform. The green LED light attached to the apparatus emits light into the glass plate, and this light is only refracted wherever rodent paws contact the glass, allowing the high speed digital camera to capture precise rodent paw placement in real time. The overhead red light creates contrast for recording of the body outline. The visual data is digitized and transferred to an attached computer where the CatWalk™ XT software can be used for semi-automated labeling and analysis of static and dynamic gait kinematics via distance, time, and intensity differences between paw prints. Gait data can then be exported for data storage and subsequent analyses.

**Table 1:** Number of animals per group according to genotype and age

	Males			Females			Total		
Age (months)	4	10	17	4	10	17	4	10	17
Gclm^+/+^	15	15	15	13	15	9	28	30	24
Gclm^-/-^	15	15	8	14	12	12	29	27	20

The alley was adjusted to be 8 cm wide, and the walkway for data recoding was defined at 8 cm x 32 cm which allowed 4 full step cycles in the center of the alley. Visual scaling was calibrated prior to each use. On testing days, animals are transferred from their home cages into a polycarbonate carrier and brought into the pitch black room to acclimate for 10 minutes prior to testing. A single animal was placed onto the platform and allowed to cross the defined walkway up to 20 times. Each crossing of the platform is called a run and non-compliant runs were defined as more than 60% speed variation within a run or longer than 5 seconds. From the pool of compliant runs, only runs with less than 10% speed variation between runs were used for further analyses. The mice were not given a formal training to the apparatus, and if they stopped mid-run and/or required auditory stimulation to move, that run was not included in the final analyses as it did not reach our a-priori set criterion for compliant runs. We selected five variables that are similar in humans and relevant to human aging (Table 2), including gait speed, base of support, stride length, swing speed, and step cycle duration. All measures besides gait speed were analyzed separately for front and hind paws.

### Statistical Analyses

A three-way analysis of covariance (ANCOVA) was run with Sex, Genotype and Age as between-group factors and Body Weight as the covariate to ensure that body weights were not responsible for driving the main effects. Body weights and the various gait measures were compared using three-way analyses of variance (ANOVA) with Sex, Genotype and Age as between-group factors. Following significance of either a main effect or interaction, individual comparisons between different Sex, Genotype, or Age were performed using a single degree-of-freedom F test involving the error term from the overall ANOVA. The individual relationships between various gait measures with gait speed were determined via Pearson’s or logarithmic correlations. The *α* level was set at 0.05 for all analyses. The software used for the analyses was Systat 13 (Systat Software Inc., San Jose, CA, USA).

**Table 2:** Gait variable definitions

Gait speed	Rate of body movement in cm/s
**Base of support**	Width between the two front or two hind paws in cm
**Stride Length**	Distance between subsequent placements of the same paw for the two front or two hind paws in cm
**Swing Speed**	Rate of movement of a paw during the swing phase for the two front or two hind paws in cm/s
**Step Cycle Duration**	Time to go through both the stand and swing phases for the two front or two hind paws in s

**Figure 1.**
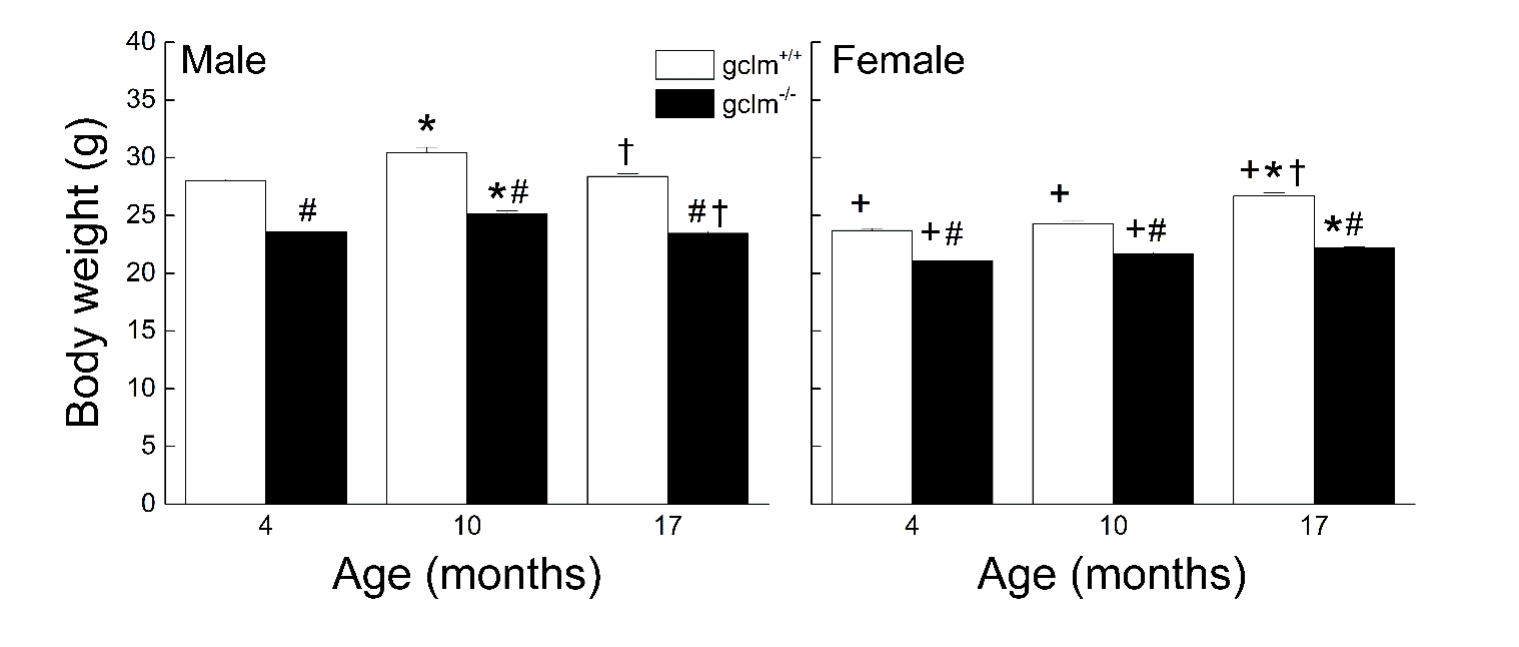
Effects of age, sex and genotype on body weights (g) in young (4 month), adult (10 month), and old (17 month) gclm^+/+^ and gclm^-/-^ mice Each value represents the mean + SEM. ^+^ p<0.05 compared to age and genotype-matched males; ^*^p < 0.05 compared to genotype-matched young; †p<0.05 adult compared to genotype-matched old; ^#^p < 0.05 compared to age-matched gclm^+/+^.

## RESULTS

### Body Weight

The effects of sex, age and genotype on the body weights are presented in Fig. 1. Overall, the males weighed more than the females regardless of genotype (13%, 17% and 5% difference in young, adult, and old respectively). The gclm^-/-^ weighed less than the gclm^+/+^ regardless of age (11-17% difference). The weight difference was more pronounced between 4 and 10 months in males, and between 10 and 17 months in females. A three-way ANOVA yielded a significant interaction between Sex, Age and Genotype supporting these observations (*p* = 0.015).

**Figure 2.**
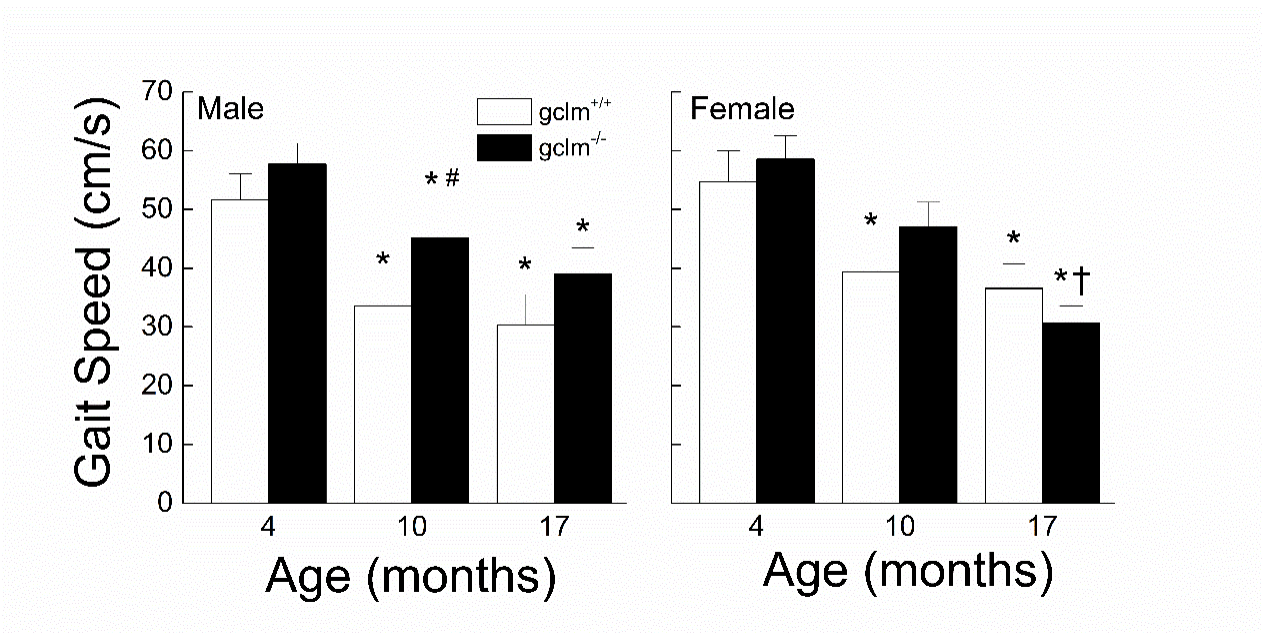
Effects of sex, age and genotype on gait speed (cm/s) in young (4 month), adult (10 month), and old (17 month) gclm^+/+^ and gclm^-/-^ mice Each value represents the mean + SEM. ^*^p < 0.05 compared to genotype-matched young; †p<0.05 adult compared to genotype-matched old; ^#^p < 0.05 compared to age-matched gclm^+/+^.

**Figure 3.**
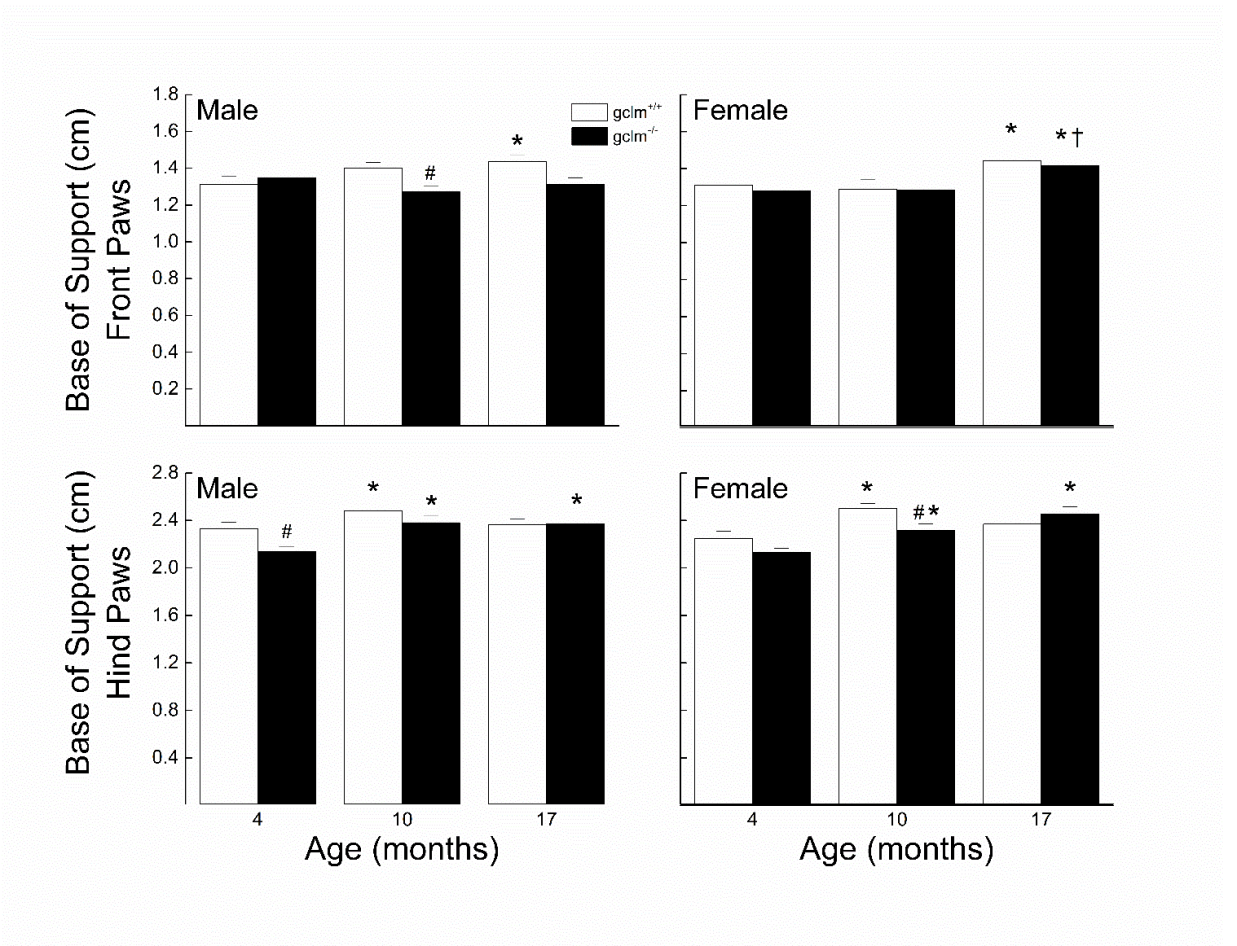
Effects of sex, age and genotype on width of the front and hind paw base of support (cm) in young (4 month), adult (10 month), and old (17 month) gclm^+/+^ and gclm^-/-^ mice Each value represents the mean + SEM. ^*^p < 0.05 compared to genotype-matched young; †p<0.05 adult compared to genotype-matched old; ^#^p < 0.05 compared to age-matched gclm^+/+^.

### Gait Speed

The effects of sex, age and genotype on the gait speed are presented in Fig. 2. There was no major difference in speed between males and females at any age. While the gait speed in both the gclm^+/+^ and gclm^-/-^ mice declined with age (gclm^+/+^: 41% in males, 33% in females; gclm^-/-^: 32% in males, 48% in females), the rate of decline was different between the two genotypes. In the gclm^+/+^ mice, the majority of the age-related decline in speed occurred between 4 and 10 months (35% for males, 28% for females), whereas in the gclm^-/-^ mice the decline was more gradual between 4 and 10 months and between 10 and 17 months. However, while males and females gclm^-/-^ declined similarly between 4 and 10 months (~21%), the decrease in speed between 10 and 17 months was more than double in the females (35%) than in the males (14%). A three-way ANOVA revealed significant main effects of Age (*p* < 0.001) and Genotype (*p* = 0.032), but there was no main effect of Sex or interactions between Sex, Age and Genotype (all *ps* ≧ 0.162).

### Base of Support

The effects of sex, age and genotype on the front and hind paw base of support are presented in Fig. 3. In males, the front and hind paws base of support widened by 10% in gclm^+/+^, which occurred mostly between 4 and 10 months. The hind paws base of support for the gclm^-/-^ was widened by 11% by 10 months along with a narrowing of 6% for the front paws base of support. Additionally, the base of support was narrower by 9% in the gclm^-/-^ at 10 and 17 months for the front paws and by 8% at 4 months for the hind paws. In gclm^+/+^ females, there was a widening of the base of support for the front paws by 10% which occurred between 10 and 17 months, while the 11% widening of the base of support for the hind paws occurred between 4 and 10 months, followed by a small narrowing of 5% between 10 and 17 months. The widening of the front paws base of support for the gclm^-/-^ was similar to that of the gclm^+/+^, while there was a gradual widening of the hind paws base of support for the gclm^-/-^. The hind paws base of support was about 6% narrower in the gclm^-/-^ than the gclm^+/+^ at 4 and 10 months. For the front paws base of support, a three-way ANOVA revealed a significant main effect of Age (*p* = 0.01), but there was no main effect of Genotype (*p* = 0.084) or any interaction between Sex, Age and Genotype (all *ps≧* 0.224). For the hind paws base of support, a three-way ANOVA revealed significant main effects of Age (*p* < 0.001) and Genotype (*p* = 0.011) and interaction of Age x Genotype (*p* = 0.028).

**Figure 4.**
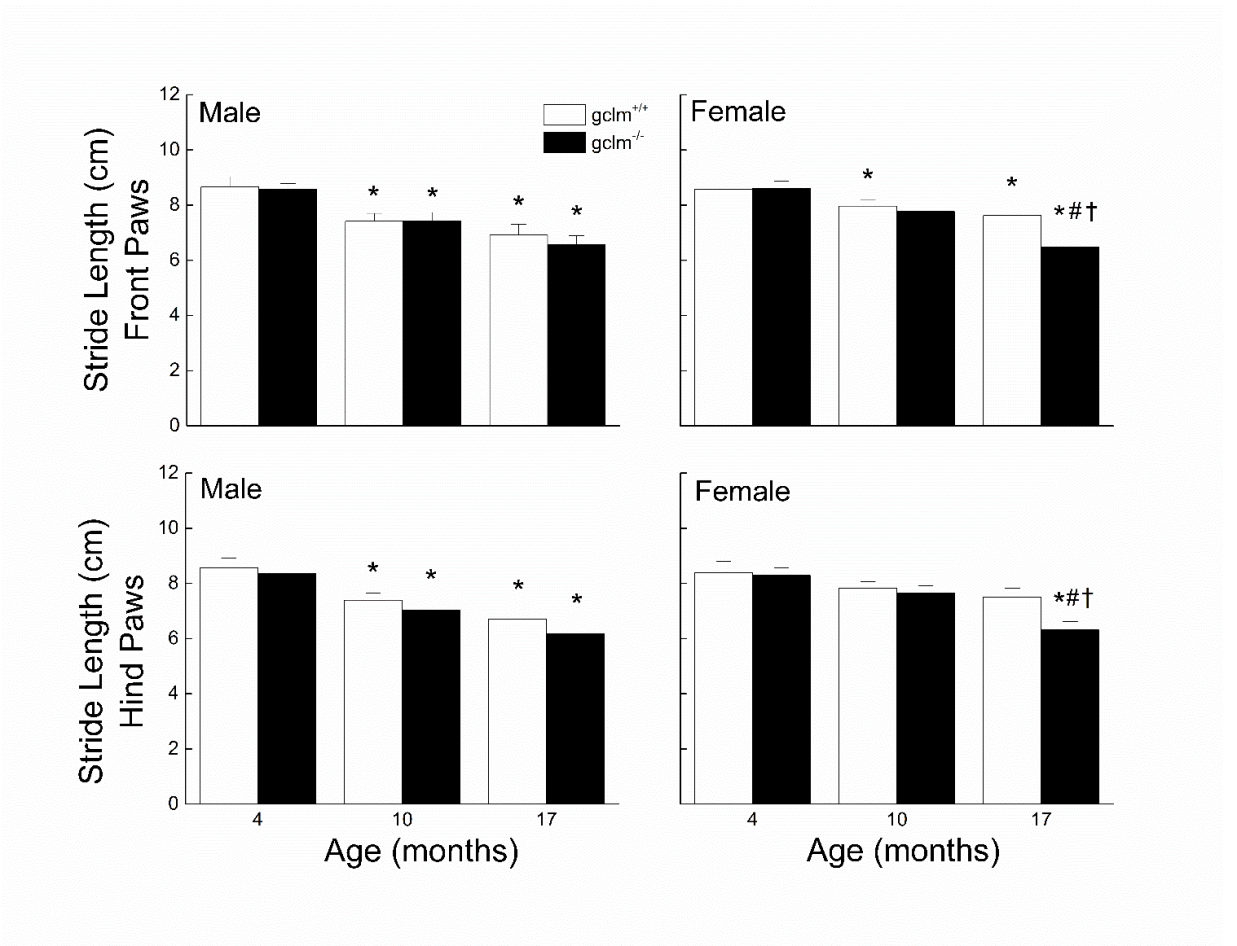
Effects of sex, age and genotype on the front and hind paw stride length (cm) in young (4 month), adult (10 month), and old (17 month) gclm^+/+^ and gclm^-/-^ mice Each value represents the mean + SEM. ^*^p < 0.05 compared to genotype-matched young; †p<0.05 adult compared to genotype-matched old; ^#^p < 0.05 compared to age-matched gclm^+/+^.

### Stride Length

The effects of sex, age and genotype on the front and hind paw stride length are presented in Fig. 4. In males, the front and hind paws stride length in both gclm^+/+^ and gclm^-/-^ mice decreased by 14% at 10 months, and further decreased by another 7-12% at 17 months. There was no difference between the gclm^+/+^ and gclm^-/-^ mice. In females, the gclm^+/+^ mice exhibited a gradual decrease in stride length reaching 10% for both front and hind paws. While the shortening of the stride was similar in both genotypes between 4 and 10 months, it was 4 times higher between 10 and 17 months in the gclm^-/-^ compared to the gclm^+/+^. At 17 months, the female gclm^-/-^ exhibited stride length for either paws 15% shorter than that of the age-matched gclm^+/+^. For the front paws, a three-way ANOVA revealed a significant main effect of Age (*p* < 0.001) but there was no main effect of Genotype (*p* = 0.125) or Sex (*p* = 0.201) or any interactions between Sex, Age and Genotype (all *ps ≧* 0.265). For the hind paws, a three-way ANOVA revealed significant main effects of Age (*p* < 0.001) and Genotype (*p* = 0.02), but there was no main effect of Sex (*p* = 0.112) or interaction of Sex, Age and Genotype (all *ps* ≧ 0.237).

### Swing Speed

**Figure 5.**
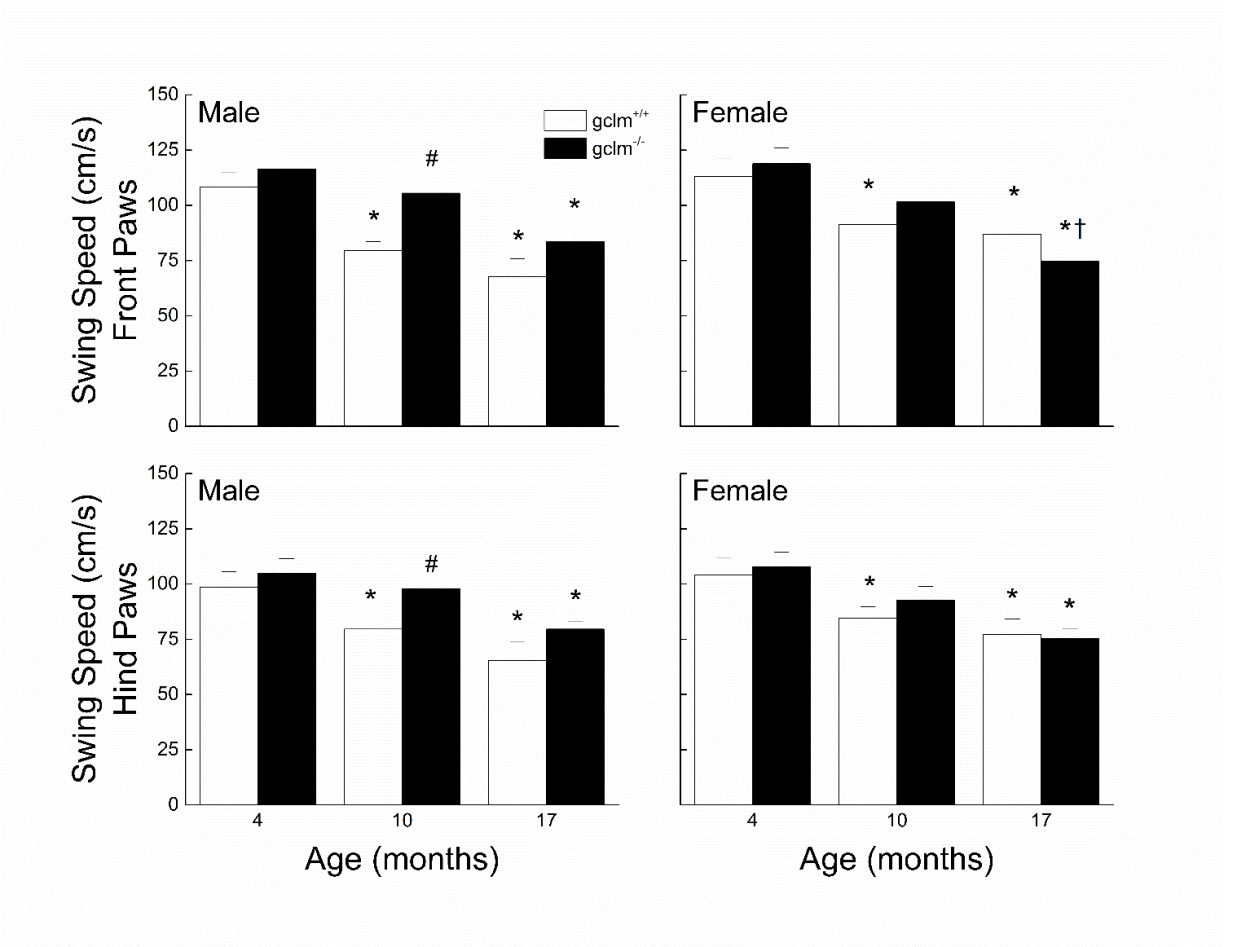
Effects of sex, age and genotype on the front and hind paw swing speed (cm/s) in young (4 month), adult (10 month), and old (17 month) gclm^+/+^ and gclm^-/-^ mice Each value represents the mean + SEM. ^*^p < 0.05 compared to genotype-matched young; †p<0.05 adult compared to genotype-matched old; ^#^p < 0.05 compared to age-matched gclm^+/+^.

The effects of sex, age and genotype on the front and hind paw swing speed are presented in Fig. 5. In males, the swing speed for both hind and front paws declined by ~36% in glcm^+/+^ and by 26% in gclm^-/-^. While the decline was gradual in the gclm^+/+^, most of the decrease occurred between 10 and 17 months in the gclm^-/-^. The swim speed of the gclm^-/-^ was higher than that of the gclm^+/+^ at 10 (front paws: 32%; hind paws: 22%) and 17 months (~22% for both front and hind paws), though it was only statistically significant at 10 months. In females, the swing speed for both hind and front paws declined by ~25% in glcm^+/+^ and by ~34% in gclm^-/-^. Contrary to the males, the decline was gradual in the gclm^-/-^ while most of the decrease occurred between 4 and 10 months in the gclm^+/+^. For both front and hind paws, three-way ANOVAs revealed significant main effects of Age (all *ps* < 0.001) and Genotype (all *ps* ≤ 0.035), however there was no main effect of Sex (all *p*s > 0.305) or interaction of Sex, Age and Genotype (All *ps* ≥ 0.064).

### Step Cycle Duration

**Figure 6.**
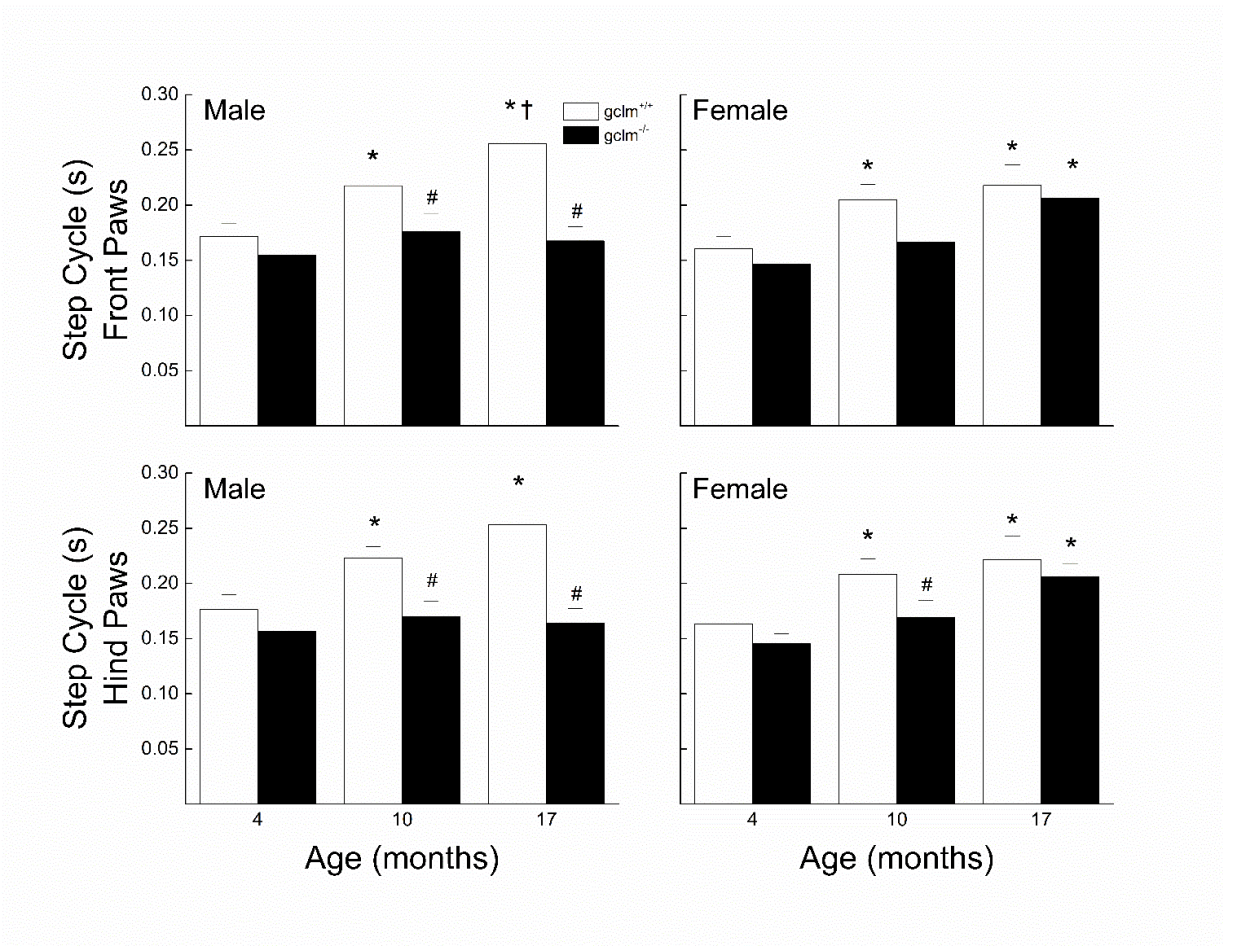
Effects of sex, age and genotype on the front and hind paw step cycle duration (s) in young (4 month), adult (10 month), and old (17 month) gclm^+/+^ and gclm^-/-^ mice Each value represents the mean + SEM. ^*^p < 0.05 compared to genotype-matched young; †p<0.05 adult compared to genotype-matched old; ^#^p < 0.05 compared to age-matched gclm^+/+^.

The effects of sex, age and genotype on the front and hind paw step cycle duration are presented in Fig. 6. In males, step cycle duration for both front and hind paws increased with age in the gclm^+/+^ by ~ 45% while it remained largely unaffected in the gclm^-/-^. The step cycle duration of the gclm^-/-^ was similar to that of the gclm^+/+^ at 4 months, but was 20% and 34% lower at 10 and 17 months, respectively. In females, both genotypes exhibited an increase in step cycle duration for both front and hind paws by 36-40%. The observed deficit was fairly gradual in the gclm^-/-^ but occurred mostly between 4 and 10 months in the gclm^+/+^. At 10 months, the gclm^-/-^ had a step cycle duration 18% shorter than that of the gclm^+/+^. Three-way ANOVAs for front and hind paws step cycle duration revealed significant main effects of Age (all *ps* < 0.001) and Genotype (all *ps* < 0.001), but there was no interaction of Sex, Age and Genotype (all *ps* ≥ 0.19) even though Sex x Genotype approached significance (*p* = 0.064 (front paws), *p*= 0.088 (hind paws)).

### Relationship of gait kinematics to gait speed

**Figure 7.**
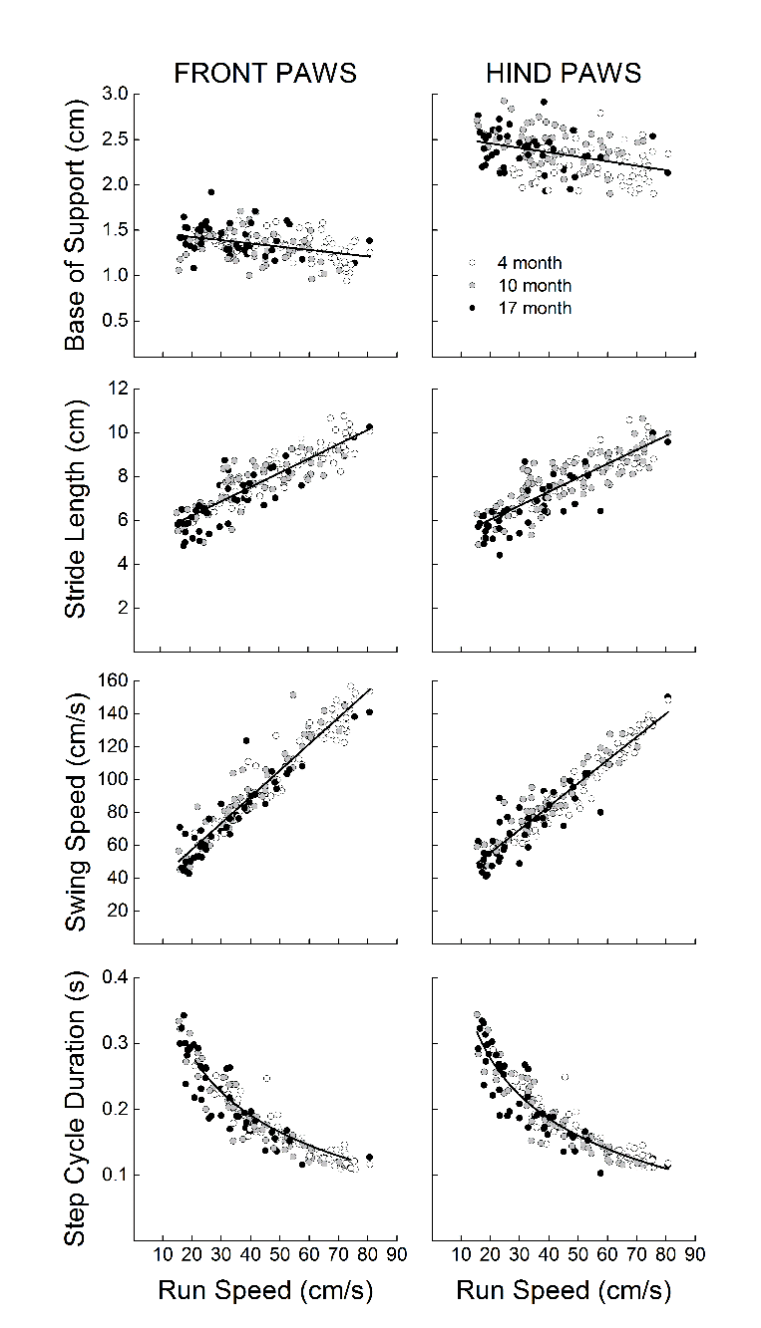
Relationship of speed with base of support, stride length, step cycle duration, and swing speed in the front and hind paws of young (4 month), adult (10 month), and old (17 month) gclm^+/+^ and gclm^-/-^ male and female mice Each value represents a single animal.

The relationships of gait speed with front and hind base of support, stride length, step cycle duration, and swing speed are presented in Fig. 7 (since there were no differences between males and females, these analyses use the combined dataset). Front paw and hind paw stride length and swing speed increased as a function of gait speed. Pearson’s correlation revealed significant relationships with gait speed for front paw (r = 0.86, *p* < 0.01) and hind paw stride length (r = 0.83, *p* < 0.01), as well as for front paw (r = 0.95, *p* < 0.01) and hind paw (r = 0.95, *p* < 0.01) swing speed. Front paw and hind paw base of support decreased as a function of gait speed. Pearson’s correlations revealed significant linear relationships of both front paw (r = -0.31, p < 0.01) and hind paw (r = -0.35, *p* < 0.01) base of support with gait speed. Front paw and hind paw step cycle duration decreased as a curvilinear function of gait speed. Polynomial regression revealed significant curvilinear relationships of both front paw (r^2^ = 0.88, *p* < 0.01) and hind paw (r^2^ = 0.87, *p* < 0.01) step cycle duration with gait speed.

## DISCUSSION

The main findings of this study were that (1) aging is associated with a slowing of gait speed, swing speed, and step cycle duration, a widening of base of support, and a shortening of the stride length, (2) glutathione deficiency delayed some of these age-related declines, with the delays being more pronounced in males, and (3) a strong relationship between all the gait variables and gait speed exists.

While CatWalk measured gait changes have been used to determine or predict function in rodent disease models such as spinal cord and brain injury, Parkinson’s disease, Huntington’s disease, amyotrophic lateral sclerosis, and arthritic damage [16-20], there remains a lack of research on quantitative gait changes as a result of aging alone. The CatWalk is a novel tool in preclinical aging research, allowing for robust quantitative measurement of rodent gait and analysis of changes across the lifespan. Most studies of aged mice have utilized measures of gait such as gait speed and function in the open field [12] or treadmill-based systems that require animals to move at set speeds [29]. While these techniques have been previously published, each has its own limitations, such as manual scoring of weight distribution, gait coordination, or stepping in the open field which leads to substantial inter and intra-observer variability, set speed of the treadmill which artificially mandates a gait speed rather than allowing the animals to move at their preferred gait speed, and ink smearing for painted foot print analysis which makes quantification difficult [16], and all lack gait kinematics analyses.

While treadmill-based gait analyses objectively quantify the same variables as the CatWalk, gait results are not equivalent between free movement and treadmill-based systems as there are known differences in gait mechanics used for the respective apparatus [14, 15]. Furthermore, Wooley *et al*. have demonstrated that the primary factor affecting gait results on the treadmill in mice is body size [4]. They later performed a predictive regression analysis based on body size (weight) for animals aged 1 - 12 months, however at 18 months animals did not fit into this trend, showing changes opposite the expected outcomes due to a decrease in body size. These results indicate that changes observed at 18 months are likely not dependent on body size changes and rather hint at age-specific gait changes [29]. Therefore at a set speed, which is the procedure for treadmill-based measurements, larger mice will have changes in various gait measures as they physically have longer legs and wider bodies. To determine whether body weight was a contributing factor to the age-related changes in gait in our study, we ran an ANCOVA on each measure with body weight as the covariate and all age effects remained indicating that body weight was not a confounding factor in our analyses of gait (all *p*s > 0.1 for age effect). Overall, the CatWalk method allows for freely-moving animals to move at their preferred speed, objectively measures many gait variables within individual runs, allows for instantaneous data analysis through digitization, thereby improving preclinical measurements of gait.

Age-related deficits in movement speed, stride length, base of support, step cycle duration and swing speed were observed at both 10 and 17 months. The gradual slowing and alteration in kinematic gait variables in the aged mice is similar to that observed with aging in humans, primarily a decline in gait speed, shortening of stride length, and a wider base of support [30]. These strategies that the elderly use are to reduce fall risk by limiting vertical center of mass displacement and increasing dynamic stability, leading to an overall less destabilizing gait pattern [31-34]. Static and dynamic balance is dependent upon the vestibular, visual and somatosensory systems as well as overall strength, and these systems have been shown to decline with age in both humans [35-37] and rodents [2, 38-41]. As a result of these changes with age, both rodents and humans employ similar adaptive strategies in gait speed and kinematics. The similarities in age-related declines in balance and gait adaptation between the two species strengthen the adequacy of the use of rodents for pre-clinical assessments of gait during aging.

In addition to the novel use of the CatWalk™ XT to examine age-related gait changes in mice, we wanted to examine potential effects of redox impairment on gait measurements. Of note, gclm^-/-^ mice exhibited age-related decline across most variables, but the age-related impairments seemed to be delayed compared to the gclm^+/+^ mice. However, the results of the ANCOVA determined that body weight was a confounding factor in the genotype differences we observed, as including weight as a covariate did not yield any significant main effects of Genotype. It is noteworthy that in the gclm^-/-^ mice step cycle duration was largely unaffected by age and swing speed declines were delayed after 10 months. While our data confirmed previous studies done in 3-5 months old mice that reported no declines in motor function of gclm^-/-^ mice [42, 43], literature evidence of effects in the old mice is non-existent. Previous studies have demonstrated that increased oxidative damage in the cerebellum with age was correlated with a worsening of motor function and balance [44]. Furthermore, acute GSH deficiency leads to impaired motor function and balance in young rodents, worsens motor neuron decline in disease models, and disrupts redox signaling in young and old rodents [45-47]. Based on the literature and our hypothesis, we expected that chronic GSH deficiency across the lifespan would lead to an accelerated pattern of functional declines, including gait measures, however, our results do not support our original hypothesis as the gclm^-/-^ mice exhibited age-related declines similar to gclm^+/+^. It is possible that the endogenous antioxidant defense or beneficial redox signaling pathways have compensated for the large deficiency in GSH and concurrent shift in redox state, and further research is warranted to pursue this hypothesis.

Sex has been implicated as a variable of interest due to potential sexual dimorphism in gait mechanics [4, 29]. Our results do not support sex differences in gait, as none of our variables had main effects or interactions with sex (all p values > 0.112), which remained even once body weights were used as a covariate. Several other studies that examined both sexes have also reported no sex differences in CatWalk-tested mice [48] or ink-tested freely-moving mice [49]. Other studies have simply combined sexes without explicit statistical justification [50]. Alternatively, sex differences in some gait measures were observed in studies using treadmill-based systems [4, 29], and could be related to differences in body size at a set speed. Additionally, sex-dependent responses in motor and gait decline in transgenic mouse models of ALS have been observed [29, 51], suggesting a differential response to both redox state and disease. There are few studies examining age-related functional decline that report the use of both sexes, but those that do have combined sexes or noted a lack of sexual dimorphism in these aged but otherwise healthy animals [52, 53], although one group examining overall frailty rather than motor function reported sex differences in their very old cohort (28 months) [54]. Including both sexes when collecting mouse data across many aspects of functional testing is generally lacking, and warrants further research to determine if both sexes age differentially in motor function and gait.

Previous studies have determined that more than 90% of the 162 gait variables measured via the CatWalk are dependent on speed [55], which we confirmed for our variables through correlation and regression testing (Fig. 7). In the context of a disease model at a set age, it is important to not confound test results that are dependent on the disease state with changes simply due to speed variance between animals. In our case, we were primarily interested in the age-related slowing of gait, and as such we did not seek to control for speed between animals [55]. By allowing speed variation we were able to capture the natural age-related slowing across the lifespan, which would otherwise be lost in a treadmill-based system. Importantly, the gait signature in relation to speed is similar between mice and humans, and hind paw gait measures have been used to compare gait dysfunction between mice and humans [56]. Additionally, the normal and maximal walking speeds both decline with age in humans [57], and this gait speed decline has previously been correlated with a shorter life expectancy [6].

### Conclusion

The associative nature of gait speed with overall health that is found in aging humans could be found to a similar extent in rodents, allowing for an additional functional outcome measure in preclinical aging studies or similar predictions of mortality, disability, or disease used in humans to be used in future mice models. Our study determined that the CatWalk is a useful tool in aging research, allowing for robust quantitative measurement of rodent gait, and analysis of changes across the lifespan. Overall, mice appear to undergo similar changes to humans, namely a “slowing” of gait with concurrent changes in gait measures. Interestingly, chronic glutathione deficiency had no detrimental effects but rather partially and mildly delayed some age-related deficits. These results warrant further examination to determine if alternative redox compensation is occurring, but the current data appears to not support the redox stress theory of aging.
